# Life‐history responses of a freshwater rotifer to copper pollution

**DOI:** 10.1002/ece3.7877

**Published:** 2021-07-29

**Authors:** Federica R. Schanz, Stefan Sommer, Andrea Lami, Diego Fontaneto, Arpat Ozgul

**Affiliations:** ^1^ Department of Evolutionary Biology and Environmental Studies University of Zurich Zurich Switzerland; ^2^ Water Research Institute National Research Council of Italy Verbania Pallanza Italy

**Keywords:** copper, life history, pollution, population growth, rotifer, vital rate

## Abstract

In organisms with dormant stages, life‐history responses to past pollution can be studied retrospectively. Here, we study such responses in a rotifer (*Brachionus calyciflorus*) from the once heavily copper‐polluted Lake Orta (Italy). We extracted resting eggs from sediments, established clonal lineages from hatchlings, and exposed newborns of these lineages to one of three copper concentrations that each mimicked a specific period in the lake's pollution history. For each rotifer, we daily collected life‐table data. We then estimated treatment‐specific vital rates and used a stage‐structured population model to project population growth rate *λ*. We also estimated elasticities of *λ* to vital rates and contributions of vital rates to observed Δ*λ* between copper treatments. As expected, *λ* decreased with increasing copper concentration. This decrease resulted mostly from a decline in juvenile survival rate (*S_J_*) and partly from a decline in the survival rate of asexually reproducing females (*S_A_*). Maturation rate, and with one exception fecundity, also declined but did not contribute consistently to Δ*λ*. *λ* was most elastic to *S_J_* and *S_A_*, indicating that survival rates were under stronger selection than maturation rate and fecundity. Together, our results indicate that variation in juvenile survival is a key component in the rotifers’ copper response. The consistent decrease in *S_J_* with increasing copper stress and the sensitivity of *λ* to that decrease also suggest that juvenile survival is a useful indicator of population performance under environmental pollution.

## Graphical abstract

We studied how rotifer vital rates respond to increasing copper pollution and whether these responses alter population fitness. We identified juvenile survival as the key component in the rotifers' copper response: juvenile survival declined with increasing copper pollution, and population fitness was sensitive to that decline.

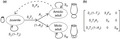

## 1 INTRODUCTION

Anthropogenic pollution of aquatic ecosystems is a global problem of increasing concern (Wilhelm, 2009). Aquatic organisms are particularly vulnerable to pollution because they absorb and ingest pollutants directly from the environment (Young et al., 2016). Individuals that are unable to escape pollution may respond quickly via phenotypic plasticity; and populations may respond slowly via genetic change (Lopes et al., 2004). However, because rates of genetic change are often outpaced by rates of anthropogenic pollution (Wilhelm, 2009), the fate of populations may largely depend on the individuals’ plastic response. Here, we investigate such a response in a rotifer (*Brachionus calyciflorus* Pallas, 1776) from a subalpine lake with a well‐documented history of intense anthropogenic pollution, Lake Orta in northern Italy (Bonacina, 2001).

Lake Orta was once heavily polluted by local industry and now represents an ideal ecosystem for studying organisms’ life‐history responses to pollution (Sommer, Nandini, et al., 2016). The pollution started in 1926 from copper‐ and ammonium‐sulfate contaminated wastewater, leading to a steady increase in copper in the lake (Bonacina, 2001). By the late 1950s, the copper concentration had reached 108 µg Cu L^–1^ (Piscia et al., 2012). Because copper was removed from the wastewater thereafter, the copper concentration halved within a decade but leveled off at about 35 µg Cu L^–1^ by the mid‐1980s. By that time, the lake had acidified to an extent that further recovery required liming. The spreading of powdered limestone in 1989 and 1990 neutralized the lake water within a decade (Bonacina, 2001). Copper and other heavy metals precipitated, and the water quality returned to prepollution conditions (Rogora et al., 2016).

Only a few organisms were able to live in the increasingly polluted lake from the late 1920s onwards (Bonacina, 2001). Brachionid rotifers were particularly successful (Rogora et al., 2016), not least because of their life cycle. The brachionid life cycle comprises sexual (mictic) and asexual (amictic) phases (Gilbert, 2003). Amictic females produce diploid eggs that hatch directly into female offspring. Mictic females, by contrast, produce haploid oocytes that either hatch directly into males or, if fertilized, develop into female embryos that quickly enter diapause. These so‐called resting eggs do not hatch immediately but are shed by the female and often sediment. Once embedded in sediment, they may survive for decades (Piscia et al., 2012). Viable resting eggs can then be resurrected in the laboratory, making brachionid rotifers excellent organisms for studying pollution responses retrospectively (Sommer, Nandini, et al., 2016). An earlier study conducted on Lake Orta brachionids did not reveal any evidence of adaptive evolution to copper exposure in these rotifers (Zweerus et al., 2017).

Rotifers are key components of aquatic food webs and important organisms in pollution monitoring and ecotoxicological testing (Wallace, 2002). In the latter context, brachionid rotifers in particular have been used to investigate effects of common pollutants on the behavior, morphology, and life history of either isolated individuals or small populations (Snell & Janssen, 1995). In *Brachionus calyciflorus*, one of the most widely studied rotifer species (Dahms et al., 2011), copper has been reported to impair swimming performance, feeding rate, body growth, and a range of demographic parameters including survival, fecundity, and consequently population growth (Charoy et al., 1995; Ferrando et al., 1993; Gama‐Flores et al., 2007; Janssen et al., 1993; Janssen, Ferrando, et al., 1994, Janssen, Persoone, et al., 1994; Snell & Moffat, 1992; Snell et al., 1991). A comparison across studies further indicated that individual‐level characteristics, such as survival and fecundity, are less sensitive to copper than population‐level characteristics such as the intrinsic rate of natural increase *r* (Preston & Snell, 2001). This conclusion, however, is compromised by varying copper concentrations and exposure times across the studies. Moreover, how strongly individual‐level effects link with population‐level consequences is still unknown.

The links between individual‐level effects of toxicants and their population‐level consequences can be studied by performing a life‐table response experiment (LTRE; Caswell, 1996a, 2000a). In such an experiment, the treatment consists of different toxicant concentrations. The individual‐level effects of the toxicant are then measured through the responses of the vital rates (survival, development, reproduction); and the population‐level consequences of these responses are assessed using demographic summary statistics such as the asymptotic population growth rate *λ* ( = *e^r^*; Caswell, 1989). The response of *λ* to the toxicant can then be decomposed into individual contributions of the vital rates to that response. Noteworthy, LTRE results are suited to project population growth assuming a constant environment, but they are unsuited to predict population growth in a changing environment (Caswell, 1996a).

Here, we study the links between individual‐ and population‐level copper effects in *B. calyciflorus* lineages resurrected from Lake Orta sediments. We ask, how do individual vital rates like survival, maturation, and fecundity respond to increasing copper pollution? How does population growth rate *λ* respond to increasing copper pollution? How elastic is *λ* to changes in vital rates? And how much does each vital rate contribute to the copper response of *λ*? As to the first two questions, we expect all vital rates, and consequently *λ*, to decline with increasing copper stress (cf. literature cited above); as to the last two questions, we have no a priori expectations. We address these questions in a LTRE involving different copper treatments. Based on the life‐table data, we first estimate treatment‐specific vital rates and employ a stage‐structured matrix population model to project *λ* (Caswell, 2001). Using prospective and retrospective perturbation analyses (Caswell, 2000b), we then estimate elasticities of *λ* to vital rates and LTRE contributions of vital rates to observed Δ*λ* between copper treatments, respectively. Together, these analyses allow us to pinpoint the key vital rates affecting population fitness under copper stress.

## 2 METHODS

### 2.1 Pre‐experimental procedures

The study organism *Brachionus calyciflorus* represents a globally distributed complex of cryptic species (Papakostas et al., 2016). The resident species in Lake Orta has been identified as *Brachionus calyciflorus* sensu stricto (Michaloudi et al., 2018). We resurrected resting eggs from three copper‐pollution periods: the mid‐1950s to mid‐1960s (> 70 µg Cu L^–1^), the early 1970s to early 1980s (35–55 µg Cu L^–1^), and post‐2000 (< 3 µg Cu L^–1^). Rotifer populations from these periods are hereafter referred to as the peak‐pollution, the recovery, and the post‐pollution population, respectively. Sediment collection and resting‐egg resurrection are described in Sommer, Nandini, et al. (2016).

To start clonal stock cultures (lineages), we transferred hatchlings to individual Petri dishes (diameter: 35 mm) filled with artificial freshwater (Sommer, Nandini, et al., 2016). To promote fast growth of lineages, we fed the rotifers green algae ad libitum (3 × 10^6^ *Chlorella vulgaris* cells mL^–1^). We kept all lineages in darkness at 20 ℃ (Panasonic incubator, MIR‐154, Japan) and renewed the culture medium daily. The experiment started once the lineages had passed the F2 generation.

To obtain experimental rotifers from each lineage, we transferred egg‐carrying, amictic females individually to round, capped plastic boxes (diameter × height: 22 mm × 13 mm) filled with 1 mL of culture medium containing food ad libitum. These boxes were stored at 20 ℃ in darkness. On the following day, we randomly selected newborn females as the experimental individuals.

### 2.2 Experimental procedures

Using the capped plastic boxes, we exposed each experimental rotifer individually for the entire lifetime to either 0, 40, or 80 µg Cu L^–1^, added as copper sulfate pentahydrate, and a food density of 1 × 10^6^ algal cells mL^–1^. These copper concentrations are within the range of historic pollution levels. In water, copper sulfate dissolves into cupric ions and other labile copper species (Camusso et al., 1991). We did not measure the bioavailability of these species but assumed that the toxicity of the treatments increased with increasing copper sulfate added (Pradeep et al., 2015; Zweerus et al., 2017). That is, the experimental copper concentrations represent nominal added concentrations (De Schamphelaere et al., 2006).

**FIGURE 1:**
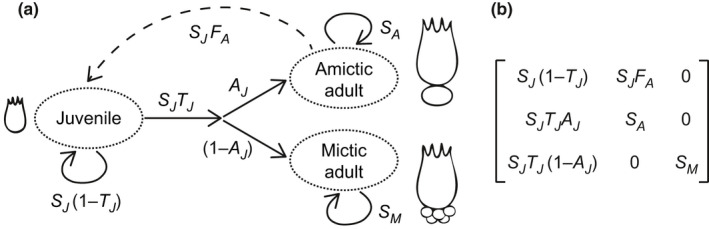
Life‐cycle diagram for *B. calyciflorus* rotifers and corresponding population projection matrix. (a) Schematic representation of the brachionid life cycle including juveniles (subscript *J*) as well as mictic and amictic adults (subscripts *M* and *A*). *S_J_*, *S_A_*, and *S_M_* are survival rates, *T_J_* is maturation rate, *A_J_* is the probability of being an amictic individual, and *F_A_* is the fecundity of amictic adults. Arrow‐headed solid and broken lines represent stage transitions and reproductive events, respectively. Note that the reproductive type (mictic or amictic) is unknown at the juvenile stage. As a consequence, juveniles are pooled into a single stage. (b) Population projection matrix derived from the life cycle

We labeled all boxes with a unique number that encoded, and masked to the data collector, each rotifer's population identity and experimental copper concentration. We kept the boxes at 20 ℃ in darkness and daily transferred the rotifers to new boxes containing fresh medium supplemented with food and the corresponding copper concentration. Before the transfer, we recorded whether a given rotifer was still alive (we discarded dead rotifers), how many female offspring it had produced (including dead ones), and whether it was mictic or amictic. The reproductive type of dead juveniles remained unknown, because mictic and amictic females can only be distinguished once they carry eggs (amictic females produce larger eggs than mictic females; Figure 1a). We collected the data for a given rotifer always at the same time of day (± 30 min). To determine whether female offspring were mictic or amicitc, we transferred them to individual boxes containing unpolluted culture medium and food ad libitum. We stored these boxes at 20 ℃ in darkness until the offspring carried eggs or died.

Because of constraints regarding workload and incubator space, we split the experiment into four runs. We extracted resting eggs before each run to synchronize the ages of stock cultures from which test individuals were taken. Because resting‐egg availability was uneven across lineages at a given time, the matching of stock‐culture ages led to an unbalanced design with respect to the number of lineages tested per run. During the first run, we tested rotifers from two peak‐pollution lineages, three recovery lineages, and one post‐pollution lineage; during the second run, we tested rotifers from four peak‐pollution lineages, three recovery lineages, and one post‐pollution lineage; during the third run, we tested rotifers from two peak‐pollution lineages and three recovery lineages; and during the fourth run, we tested rotifers from five peak‐pollution lineages and four recovery lineages. We set up three replicates for each lineage and copper treatment, resulting in 252 experimental rotifers. Because we found only two viable resting eggs in the post‐pollution sediments, we excluded this period from the analyses. The other two periods were represented by 13 lineages each, leaving life‐table data of 234 rotifers for the analyses (Schanz et al., 2021).

### 2.3 Data analysis

We performed all analyses in R, version 4.0.2 (R Core Team, 2020), using the package *lme4*, version 1.1‐23 (Bates et al., 2015). We employed a stage‐structured rather than an age‐structured life cycle, because the sensitivity to toxicants often varies among life stages (Caswell, 2000a). In rotifers, age‐ and stage‐structured analyses result in similar estimates of population growth (Sommer et al., 2019), but only the amictic stage contributes to instantaneous population growth (Figure 1a). We distinguished between juveniles, amictic adults, and mictic adults.

First, we estimated six daily vital rates for each population‐by‐treatment combination: survival rates of juveniles (*S_J_*), amictic adults (*S_A_*), and mictic adults (*S_M_*); the probability of transitioning from the juvenile to the adult stage (i.e., maturation rate *T_J_*); the probability of being an amictic individual (*A_J_*); and the number of female offspring produced per amictic adult (i.e., fecundity *F_A_*). We treated *T_J_* and *A_J_* as conditional on juvenile survival. Moreover, we estimated *A_J_* based on the reproductive type of the offspring rather than the experimental rotifers, because the experimental rotifers were not exposed to the copper treatments during the egg stage, the period during which environmental conditions (here, the copper treatments) affect the reproductive type (Gilbert, 1963). We estimated vital rates using generalized linear mixed models (Bolker et al., 2009) and either the Poisson (for *F_A_*) or the binomial (for all other vital rates) error distribution. We treated population, copper concentration, and their interaction as fixed effects and rotifer identity and run number as crossed random effects. Assuming populations were sampled before (instead of after) reproductive events, we used the product of *F_A_* and *S_J_* (instead of *S_A_*) as the contribution to the juvenile stage (Figure 1b). To test for additive and interaction effects between population and copper concentration on the vital rates, we used Akaike's information criterion corrected for small sample sizes, AICc (Burnham & Anderson, 2002). Using the vital‐rate values, we then estimated the asymptotic population growth rate *λ* as the dominant eigenvalue of the projection matrix (Caswell, 2001). We considered *λ* as a proxy for population fitness.

Next, we performed elasticity analysis to investigate how a proportional change in each vital rate *x_k_* changes *λ*; and we applied fixed‐design LTRE methods to decompose differences in *λ* into vital‐rate‐specific contributions (Caswell, 1996b, 2001). We computed vital‐rate elasticities as

(1) $$\frac{x_{k}}{\lambda }\frac{\partial \lambda }{\partial x_{k}}=\frac{x_{k}}{\lambda }\sum_{i,j}\frac{\partial \lambda }{\partial a_{\mathrm{ij}}}\frac{\partial a_{\mathrm{ij}}}{\partial x_{k}}$$

with *a_ij_* representing the matrix element in row *i*, column *j* (Figure 1b); and we decomposed differences in *λ* between pairs of experimental groups Δ*λ*_2–1_ as

(2) $${\left.\Delta \lambda_{2‐1}\approx \sum_{k}\left(x_{k2}‐x_{k1}\right)\frac{\partial \lambda }{\partial x_{k}}\right|}_{M}$$

with vital‐rate sensitivities being evaluated using the mean matrix **M** for each pairwise comparison.

Finally, to account for parameter uncertainty, we employed a nonparametric bootstrap method (Efron & Tibshirani, 1993). Based on 2,000 bootstraps, we estimated vital rates from life‐history data of 50 randomly sampled (with replacement) individuals per population and copper treatment. We used these values to estimate *λ*s, elasticities, and LTRE contributions. We also estimated the 95% confidence intervals (CIs) by computing the 2.5th and 97.5th percentiles.

## 3 RESULTS

### 3.1 Vital rates

**FIGURE 2:**
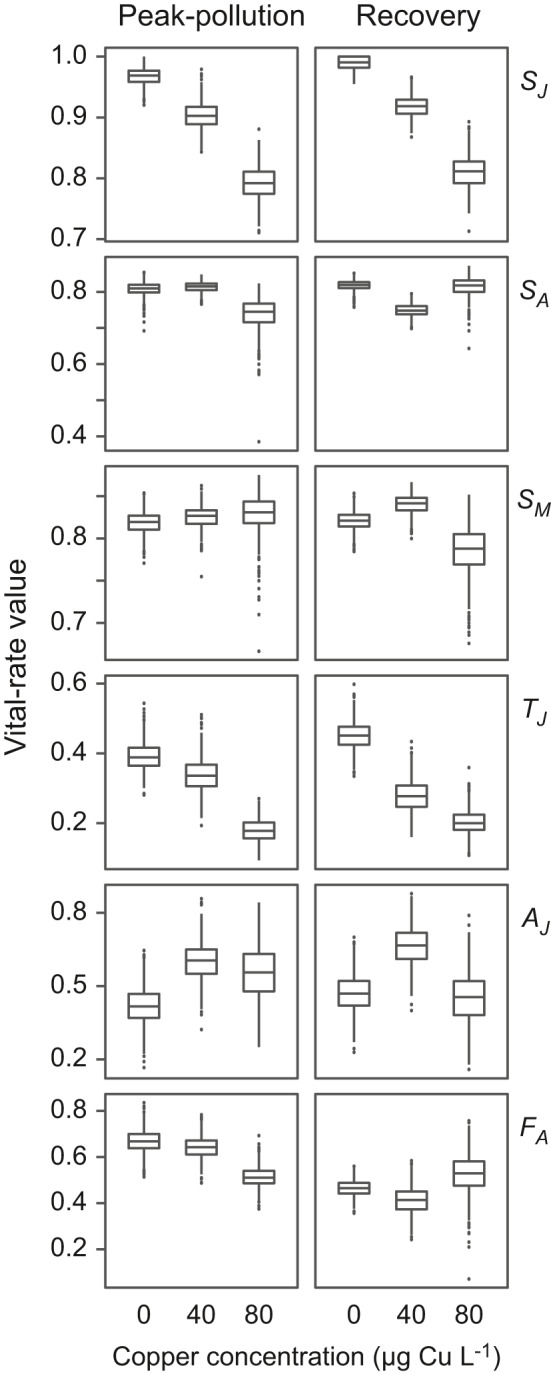
Vital‐rate estimates. Rotifers from the peak‐pollution and the recovery population were subjected to one of three copper concentrations: 0, 40, or 80 µg Cu L^–1^. Lower and upper edges of boxes depict the first and third quartiles, respectively; lines inside the boxes represent medians; whiskers extend to maximally 1.5 times the interquartile range; dots beyond whiskers indicate outliers. Survival rates *S_J_*, *S_A_*, and *S_M_* and maturation rate *T_J_* are daily rates; *A_J_* is the probability of being an amictic individual; and *F_A_* is the daily number of female offspring per amictic adult. Vital‐rate abbreviations are as in Figure 1

The six vital rates were differently affected by copper (Figure 2). Regarding juvenile survival rate (*S_J_*), the selected best model included the copper effect, but the models including an additive or an interaction effect between copper and population were within two AICc units of the selected best model (Table 1). Generally, *S_J_* decreased with increasing copper concentration and was higher in the recovery than in the peak‐pollution population (Figure 2; mean values and 95% CIs are reported in Table S1). Regarding the survival rates of amictic and mictic adults (*S_A_* and *S_M_*), the selected best models indicated neither an effect of copper nor any difference between populations (model *intercept*; Table 1). In the case of *S_A_*, the model including the copper effect was within two AICc units of the selected best model, but there was no consistent trend along the copper gradient (Figure 2; Table S1). Regarding maturation rate (*T_J_*), the selected best model included an interaction effect between population and copper, but the model including the copper effect was within two AICc units of the selected best model (Table 1). Generally, *T_J_* decreased with increasing copper concentration (Figure 2; Table S1). In the peak‐pollution population, *T_J_* decreased more steeply from 40 to 80 µg Cu L^–1^, whereas in the recovery population, it decreased more steeply from 0 to 40 µg Cu L^–1^. Regarding the probability of being an amictic individual (*A_J_*), the selected best model included an effect of copper (Table 1); *A_J_* was highest at the intermediate copper concentration (Figure 2). Finally, regarding fecundity of amictic adults (*F_A_*), the selected best model included an interaction effect between population and copper (Table 1). In the peak‐pollution population, *F_A_* decreased with increasing copper concentration, whereas in the recovery population, it did not exhibit a clear trend along the copper gradient (Figure 2).

**TABLE 1:** Results of model selection to investigate effects of population identity (*pop*) and copper concentration (*cu*) on stage‐specific vital rates of the peak‐pollution and the recovery population. *S_J_*, *S_A_*, and *S_M_* are the survival rates of juveniles, amictic adults, and mictic adults, respectively; *T_J_* is the maturation rate; *A_J_* is the probability of being an amictic individual; and *F_A_* is the fecundity of amictic adults (cf. Figure 1). Indicated for each model are the differences in Akaike's information criterion corrected for small sample sizes (ΔAICc) relative to the selected best model (ΔAICc = 0) and the degrees of freedom (*df*). The multiplication and the plus sign indicate interaction and additive effects, respectively; *intercept* represents the intercept‐only model

Model	*S_J_*	*S_A_*	*S_M_*	*T_J_*	*A_J_*	*F_A_*	*df*
*pop×cu*	0.6	9.1	11.4	0	8.6	0	11
*pop+cu*	0.2	4.4	6.9	4.9	3.9	19.5	7
*pop*	55.3	2.2	4.0	46.3	42.3	30.7	5
*cu*	0	2.0	2.9	1.0	0	37.0	5
*intercept*	54.1	0	0	42.6	38.6	41.2	3

### 3.2 Population growth rate

**FIGURE 3:**
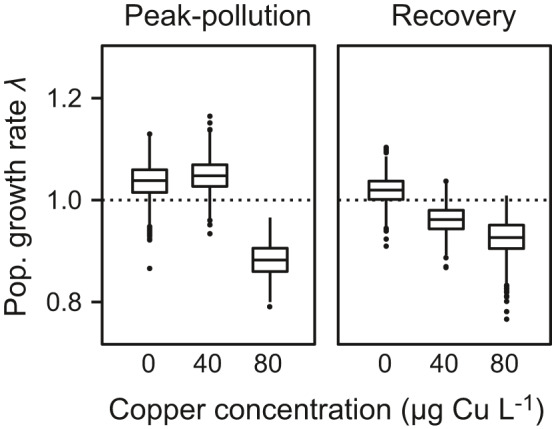
Asymptotic population growth rate *λ*. Rotifers from the peak‐pollution and the recovery population were subjected to one of three copper concentrations: 0, 40, or 80 µg Cu L^–1^. Estimates of *λ* are based on daily collected life‐table data. Values above and below the dotted line indicate positive and negative population growth, respectively. Boxplot conventions are as in Figure 2

In both populations, the asymptotic population growth rate decreased from *λ* ≈ 1 under copper‐free conditions to *λ* < 1 at the highest copper concentration (Figure 3; mean values and 95% CIs are reported in Table S1). At the intermediate copper concentration, the mean *λ* value of the peak‐pollution and the recovery population was above and below unity, respectively. However, the corresponding 95% CIs overlapped, and both intervals included *λ* = 1 (Table S1).

### 3.3 Elasticities

**FIGURE 4:**
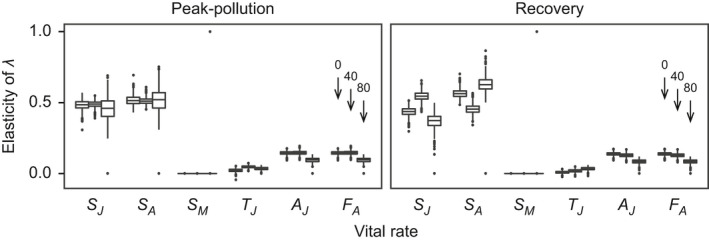
Elasticity of asymptotic population growth rate *λ*. Rotifers from the peak‐pollution and the recovery population were subjected to one of three copper concentrations. From left to right within vital rates, boxplot triplets represent treatments 0, 40, and 80 µg Cu L^–1^ (cf. arrows). Elasticity values are proportional changes in *λ* caused by proportional changes in vital rates. Vital‐rate abbreviations and boxplot conventions are as in Figures 1 and 2, respectively

Elasticity patterns were similar between populations and among treatments (Figure 4; mean values and 95% CIs are reported in Table S2). Elasticity of *λ* was highest to the survival rates of juveniles and amictic adults (*S_J_* and *S_A_*), intermediate to the fecundity of amictic adults and the probability of being an amictic individual (*F_A_* and *A_J_*), close to zero to the maturation rate (*T_J_*), and zero to the survival rate of mictic adults (*S_M_*).

### 3.4 LTRE contributions

**FIGURE 5:**
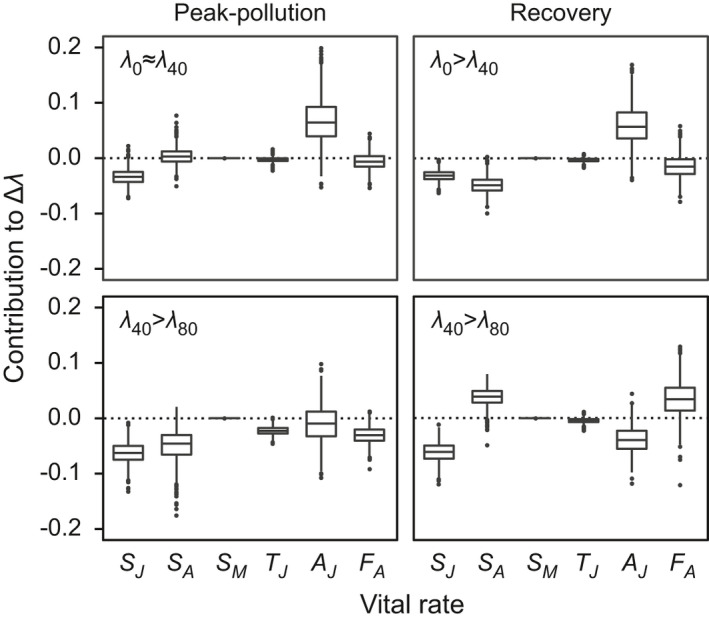
LTRE contributions to differences in asymptotic population growth rate *λ*. Rotifers from the peak‐pollution and the recovery population were subjected to one of three copper concentrations (cf. subscripts to *λ*). Shown are the contributions of each vital rate to the overall treatment effect (Δ*λ*) resulting from increasing the copper concentration from 0 to 40 µg Cu L^–1^ (top row) and from 40 to 80 µg Cu L^–1^ (bottom row). When Δ*λ* is negative (top right panel and bottom panels), negative (positive) contributions of vital rates support (oppose) Δ*λ*. Vital‐rate abbreviations and boxplot conventions are as in Figures 1 and 2, respectively

In all comparisons between copper treatments (Figure 5), differences in juvenile survival rate (*S_J_*) contributed to differences in population growth rate (Δ*λ*). None of the corresponding 95% CIs included zero (mean values and 95% CIs of LTRE contributions are reported in Table S3). In one comparison (peak‐pollution population: 40 versus 80 µg Cu L^–1^), differences in maturation rate (*T_J_*) also contributed to Δ*λ*. As for all other vital rates, the 95% CIs of the LTRE contributions included zero.

## 4 DISCUSSION

We studied how rotifer vital rates respond to increasing copper pollution and whether these responses alter population fitness. For a vital rate to alter population fitness under environmental change, and for doing so in a predictable way, two conditions must be met: the vital rate must covary with the environment; and population fitness must be sensitive to the variation in the vital rate (Benton & Grant, 1999). Here, both conditions were met only by juvenile survival rate.

Fitness consequences of copper pollution have also been studied in Lake Orta water fleas (genus *Daphnia*), which were absent from the lake water during high pollution but reappeared in the 1980s (Piscia et al., 2006). Similar to the rotifers studied here, *Daphnia* produce dormant propagules (ephippia). Piscia et al. (2015) collected individuals of the species *D. galeata* from the now unpolluted water and resurrected ephippia from sediments deposited during the 1980s. They then exposed individuals to 0, 10, and 40 µg Cu L^–1^. The resurrected *Daphnia* reproduced at all copper concentrations, whereas the contemporary *Daphnia* reproduced only in unpolluted water. Moreover, a follow‐up analysis revealed that the copper response of *D. galeata* was mostly mediated by reproductive parameters and maturation rate (Sommer, Piscia, et al., 2016). In the rotifers studied here, the copper response was mostly mediated by survival rates. In both taxa, however, population growth rate was most elastic to the survival rates of adults, followed by the survival rates of juveniles, indicating that survival is under stronger selection than maturation and fecundity (Hilde et al., 2020).

To study potential evolutionary adaptation to copper pollution, ideally one would compare copper tolerance of lineages from pre‐pollution, pollution, and post‐pollution times. Although pre‐pollution resting eggs have been found previously in Lake Orta sediments (Piscia et al., 2012), we failed to find such resting eggs in our sediment samples, and we found only two viable post‐pollution resting eggs. Nonetheless, we can compare the performances of the peak‐pollution and the recovery populations. Temporally, these populations are separated by more than a decade during which the copper concentration in Lake Orta halved. Because tolerance can be lost quickly once conditions improve (Hairston et al., 1999; Turko et al., 2016), one might expect the peak‐pollution population to outperform the recovery population, especially under the highest copper concentration. Our population growth rate estimates do not support this expectation. Only at the intermediate copper concentration, representing recovery pollution levels, the peak‐pollution population grew faster than the recovery population; at the highest copper concentration, representing peak‐pollution levels, both populations declined at a similar rate. These results suggest that Lake Orta rotifers either did not adapt genetically to contemporary copper concentrations or did adapt when the copper concentration increased but remained adapted when the copper concentration declined. Two lines of evidence support the latter explanation. First, brachionid rotifers are known to be able to adapt and survive under high copper concentrations (Aránguiz‐Acuña et al., 2018). In Lake Orta, *B. calyciflorus* rotifers produced resting eggs throughout the pollution (Piscia et al., 2012, 2016). Second, the genetic fingerprint of the mitochondrial lineages of *B. calyciflorus* in Lake Orta is identical throughout the pollution (Piscia et al., 2012), indicating that immigration from outside the lake, which could have prevented genetic adaptation, did not occur. That is, the resident population may have adapted to copper exposure and persisted in the lake without being replaced by incoming propagules, as it is expected with the strong priority effect of brachionid rotifers with a resting‐egg bank of locally adapted genotypes buffering against colonizers (De Meester et al., 2002).

The experimental populations were projected to decline at the highest copper concentration, suggesting that the natural populations should have been unable to grow in Lake Orta during the peak‐pollution years. A previous laboratory experiment supports this conclusion: population growth became negative above 30 µg Cu L^–1^ (Sommer et al., 2017). Because the copper in Lake Orta exceeded this concentration from the mid‐1930s to the late 1980s (Piscia et al., 2012), the inability of the laboratory populations to grow under these conditions suggests that the rotifers in Lake Orta should have disappeared from the water column. Yet the prevalence of resting eggs in the corresponding sediment layers indicates that active populations were present in the water column during these times (Piscia et al., 2016). The discrepancy between experimental prediction and sediment evidence likely results from environmental differences between the laboratory and the lake. Such differences include, for example, water composition, food quality and quantity, and water temperature, which all affect the toxicity of copper to the rotifers (Sarma et al., 2000; De Schamphelaere et al., 2006; Snell et al., 1991; Xu et al., 2021). However, because the experimental manipulation of copper concentration did not change the pattern of population‐growth elasticity qualitatively, we assume that the elasticity pattern might be robust to environmental change affecting copper toxicity. That is, although population growth rate at a given copper concentration likely differs between the lake and the laboratory, the key role of juvenile survival in the rotifers’ copper response might hold true in both environments.

We projected population growth rate based on vital rates realized under density‐independent conditions. Under such conditions, population growth approximates *r*_max_ (Cortés, 2016), the intrinsic rate of natural increase, which exceeds population growth realized under density‐dependent conditions. However, population growth of *r*‐selected organisms such as *B. calyciflorus* is affected mostly by vital‐rate performance accomplished at low densities (Allan, 1976). At low densities, *B. calyciflorus* females produce proportionally fewer mictic offspring than at high densities (Gilbert, 1963, 2003). Therefore, *λ* as projected here is a relevant parameter to assess the fitness of *B. calyciflorus* populations.

The experimental design prevented us from including sexual reproduction into the population fitness projections. Because individuals were kept in isolation, mictic females could not mate and therefore did not produce resting eggs (they produced males). With increasing copper concentration, mictic *B. calyciflorus* females have been reported to produce fewer resting eggs (Preston & Snell, 2001). A previous study on resurrected Lake Orta lineages reported only weak evidence for such a trend (Zweerus et al., 2017). However, in this previous study, rotifers from the peak‐pollution population produced more resting eggs than rotifers from the recovery population (Zweerus et al., 2017). This difference reflects well the prevalence of resting eggs in the corresponding sediment layers (Piscia et al., 2016); and it is in line with the hypothesis that resting eggs represent a strategy to escape pollution (Aránguiz‐Acuña & Serra, 2016). However, if resting‐egg production was a key component of the rotifers’ copper response, the proportion of mictic females should have increased with increasing copper concentration. In our experiment, such a trend did not emerge.

The aim of this study was to learn how increasing copper pollution affects rotifer vital rates and how these effects translate into changes in population growth. As expected, population growth declined with increasing pollution, and juvenile survival played a key role in this decline. This finding indicates that the widely used 24‐hr LC50 value in ecotoxicological studies (the toxicant concentration that causes 50% mortality in neonates exposed for 24 hr; reviewed in Snell & Janssen, 1995; Rico‐Martínez et al., 2013; and Won et al., 2017) is a meaningful criterion for assessing how water pollution affects the viability of rotifer populations.

## CONFLICT OF INTEREST

The authors declare no conflict of interest.

## AUTHOR CONTRIBUTION

**Federica R Schanz:** Conceptualization (supporting); Formal analysis (supporting); Investigation (lead); Methodology (equal); Software (supporting); Writing—review & editing (supporting). **Stefan Sommer:** Conceptualization (equal); Investigation (supporting); Methodology (equal); Project administration (supporting); Supervision (supporting); Writing—original draft (lead); Writing—review & editing (lead). **Andrea Lami:** Resources (supporting); Writing—review & editing (supporting). **Diego Fontaneto:** Resources (supporting); Writing—review & editing (supporting). **Arpat Ozgul:** Conceptualization (equal); Formal analysis (lead); Funding acquisition (lead); Methodology (equal); Project administration (lead); Resources (lead); Software (lead); Supervision (lead); Writing—review & editing (supporting).

## Supporting information

Supplementary Material

## Data Availability

The data are available from the Dryad Digital Repository: https://doi.org/10.5061/dryad.z08kprrcx.
