# Supplementary material for: Life‐history responses of a freshwater rotifer to copper pollution
Source: Ecol Evol. 2021 Jul 29;11(16):10947–55. doi: 10.1002/ece3.7877 (PMC8366851; doi:10.1002/ece3.7877)
Supplement: Supplementary file 1 — Supplementary Material [file ECE3-11-10947-s001.pdf]

SUPPORTING INFORMATION

TABLE S1. Vital rates and population growth rate of rotifers from the peak-pollution and the recovery population. Values represent means [95% confidence intervals]. S_J, S_A, and S_M are survival rates, T_J is maturation rate, A_J is the probability of being an amictic individual, F_A is the fecundity of amictic adults, and λ is the asymptotic population growth rate; subscripts J, A, and M refer to the juvenile, amictic-adult, and mictic-adult stage, respectively; Cu is the copper concentration (µg Cu L^{-1}).

Cu	S_J	S_A	S_M	T_J	A_J	F_A	λ
**Peak-pollution population**							
0	0.97	0.81	0.82	0.39	0.42	0.67	1.04
	[0.94, 0.99]	[0.77, 0.84]	[0.79, 0.84]	[0.32, 0.47]	[0.28, 0.57]	[0.57, 0.76]	[0.96, 1.10]
40	0.90	0.81	0.83	0.34	0.60	0.64	1.05
	[0.86, 0.94]	[0.78, 0.84]	[0.80, 0.85]	[0.25, 0.44]	[0.45, 0.74]	[0.55, 0.73]	[0.99, 1.11]
80	0.79	0.74	0.83	0.18	0.56	0.51	0.88
	[0.74, 0.85]	[0.64, 0.80]	[0.78, 0.86]	[0.12, 0.25]	[0.35, 0.76]	[0.43, 0.60]	[0.83, 0.94]
**Recovery population**							
0	0.99	0.82	0.82	0.45	0.47	0.46	1.02
	[0.97, 1.00]	[0.79, 0.84]	[0.80, 0.84]	[0.37, 0.53]	[0.33, 0.62]	[0.40, 0.53]	[0.96, 1.07]
40	0.92	0.75	0.84	0.28	0.66	0.41	0.96
	[0.89, 0.95]	[0.72, 0.78]	[0.82, 0.86]	[0.20, 0.37]	[0.51, 0.81]	[0.31, 0.52]	[0.91, 1.01]
80	0.81	0.81	0.79	0.20	0.45	0.53	0.93
	[0.76, 0.86]	[0.76, 0.85]	[0.73, 0.83]	[0.14, 0.27]	[0.26, 0.65]	[0.36, 0.68]	[0.85, 0.99]

TABLE S2. Elasticities of population growth rate to vital rates. Values represent means [95% confidence intervals]. Cu is the copper concentration (μg Cu L^{-1}); vital-rate abbreviations are as in Table S1.

**Cu**	S_J	S_A	S_M	T_J	A_J	F_A
**Peak-pollution population**						
0	0.48	0.52	0.00	0.02	0.14	0.14
	[0.41, 0.54]	[0.46, 0.59]	[0.00, 0.00]	[0.00, 0.04]	[0.12, 0.17]	[0.12, 0.17]
40	0.49	0.51	0.00	0.05	0.15	0.15
	[0.44, 0.53]	[0.47, 0.56]	[0.00, 0.00]	[0.03, 0.06]	[0.12, 0.17]	[0.12, 0.17]
80	0.42	0.47	0.01	0.03	0.09	0.09
	[0.00, 0.60]	[0.00, 0.67]	[0.00, 1.00]	[0.00, 0.05]	[0.00, 0.12]	[0.00, 0.12]
**Recovery population**						
0	0.44	0.56	0.00	0.01	0.14	0.14
	[0.37, 0.49]	[0.51, 0.63]	[0.00, 0.00]	[0.00, 0.02]	[0.11, 0.16]	[0.11, 0.16]
40	0.55	0.45	0.00	0.02	0.13	0.13
	[0.48, 0.60]	[0.40, 0.52]	[0.00, 0.00]	[0.00, 0.04]	[0.10, 0.15]	[0.10, 0.15]
80	0.37	0.63	0.00	0.03	0.09	0.09
	[0.26, 0.45]	[0.55, 0.73]	[0.00, 0.00]	[0.01, 0.05]	[0.06, 0.12]	[0.06, 0.12]

TABLE S3. Life-table response experiment contributions of vital rates to differences in population growth rate. Values represent means [95% confidence intervals]. Subscripts to λ are copper concentrations (µg Cu L^{-1}); vital-rate abbreviations are as in Table S1.

λ_{Cu1} vs. λ_{Cu2}	S_J	S_A	S_M	T_J	A_J	F_A
**Peak-pollution population**						
λ_0 vs. λ_{40}	-0.03	0.00	0.00	0.00	0.07	-0.01
	[-0.06, -0.01]	[-0.02, 0.03]	[0.00, 0.00]	[-0.01, 0.00]	[-0.01, 0.16]	[-0.03, 0.02]
λ_{40} vs. λ_{80}	-0.06	-0.05	0.00	-0.02	0.00	-0.03
	[-0.10, -0.02]	[-0.12, 0.00]	[0.00, 0.00]	[-0.04, -0.01]	[-0.07, 0.06]	[-0.06, 0.00]
**Recovery population**						
λ_0 vs. λ_{40}	-0.03	-0.05	0.00	0.00	0.06	-0.02
	[-0.05, -0.01]	[-0.08, -0.02]	[0.00, 0.00]	[-0.01, 0.00]	[-0.01, 0.13]	[-0.05, 0.02]
λ_{40} vs. λ_{80}	-0.06	0.04	0.00	0.00	-0.04	0.04
	[-0.10, -0.03]	[0.00, 0.07]	[0.00, 0.00]	[-0.01, 0.00]	[-0.08, 0.01]	[-0.02, 0.10]
